# Correlates of consistent condom use among men who have sex with men recruited through the Internet in Huzhou city: a cross-sectional survey

**DOI:** 10.1186/1471-2458-13-1101

**Published:** 2013-12-01

**Authors:** Meihua Jin, Zhongrong Yang, Zhengquan Dong, Jiankang Han

**Affiliations:** 1 Huzhou Center for Disease Control and Prevention, Huzhou 313000, Zhejiang Province, China

**Keywords:** HIV, AIDS, Men who have sex with men (MSM)

## Abstract

**Background:**

There is growing evidence that men who have sex with men (MSM) are currently a group at high risk of HIV infection in China. Our study aims to know the factors affecting consistent condom use among MSM recruited through the internet in Huzhou city.

**Methods:**

An anonymous cross-sectional study was conducted by recruiting 410 MSM living in Huzhou city via the Internet. The socio-demographic profiles (age, education level, employment status, etc.) and sexual risk behaviors of the respondents were investigated. Bivariate logistic regression analyses were performed to compare the differences between consistent condom users and inconsistent condom users. Variables with significant bivariate between groups’ differences were used as candidate variables in a stepwise multivariate logistic regression model. All statistical analyses were performed using SPSS for Windows 17.0, and a *p* value < 0.05 was considered to be statistically significant.

**Results:**

According to their condom use, sixty-eight respondents were classified into two groups. One is consistent condom users, and the other is inconsistent condom users. Multivariate logistic regression showed that respondents who had a comprehensive knowledge of HIV (*OR* = 4.08, *95% CI*: 1.85-8.99), who had sex with male sex workers (*OR* = 15.30, *95% CI*: 5.89-39.75) and who had not drunk alcohol before sex (*OR* = 3.10, *95% CI*: 1.38-6.95) were more likely to be consistent condom users.

**Conclusions:**

Consistent condom use among MSM was associated with comprehensive knowledge of HIV and a lack of alcohol use before sexual contact. As a result, reducing alcohol consumption and enhancing education regarding the risks of HIV among sexually active MSM would be effective in preventing of HIV transmission.

## Background

In recent years, dramatic increasing in human immunodeficiency virus (HIV) transmission among men who have sex with men (MSM) has been observed in China [1]. In 2009, MSM accounted for 33% of new HIV infections in China [2]. Huzhou city is a low AIDS epidemic area in China, but has rapidly increasing HIV prevalence among MSM in recent three years (up from 4.1% in 2009 to 31.7% in 2012). MSM are at high risk of becoming infected with HIV due to their sexual behaviors such as multiple sexual partners and unprotected anal intercourse (UAI, i.e., inconsistent condom use) [3]. Recently, more and more MSM have started to select their sexual partners via the Internet, and less concern has been focused on this population. Many studies have reported sexual risk factors that are associated with UAI among MSM recruited using venue-based sampling [4-6], but it is unknown whether MSM who meet sexual partners via the Internet and who use condoms inconsistently also have other risky behaviors. There is a dearth of data comparing the sexual risk factors between consistent and inconsistent condom users among MSM who meet partners through the Internet.

As an important tool, the Internet has used for the delivery of health promotion and disease prevention interventions [7-11]. Based on previous surveys that showed increasing rates of sexually transmitted diseases (STD) associated with HIV among MSM [12-14], studies aimed at investigating the potential associations between consistent condom use and risky behaviors among MSM may be valuable. Consistent condom use is an effective strategy in preventing STDs [15], and the examination of factors that influence consistent condom use among MSM is important for public health interventions. Accordingly, the purpose of our cross-sectional study was to identify associations between consistent condom use and associated risk behaviors among MSM. Our present study compared socio-cultural factors and levels of risky behaviors among MSM in Huzhou city, Zhejiang province. These respondents were recruited by Internet-based sampling methods, and they were divided into two groups (consistent condom users and inconsistent condom users) according to whether they used a condom every time they had sexual contact with both stable and casual partners. Independent variables associated with these behaviors, including socio-demographic factors, the prevalence of self-reported STD, risk behaviors and utilization of HIV prevention services, were investigated. Due to the Internet has playing an important role in spreading HIV among MSM through risky sexual behaviors [8,10,12-14], the information about consistent condom use among MSM in China recruited through the Internet is limited and our present study is well timed, so it is important for us to carry out this research.

## Methods

### Study population and data collection

This study focuses on MSM aged between 15 and 47 years who reported having anal sex with men in the last six months in Huzhou city, Zhejiang province, China. A total of 410 respondents were recruited by convenience sampling [16,17] from seven MSM-oriented Tencent QQ (internet-based instant messaging software that was developed by Shenzhen Tencent computer system Co., Ltd.) “making friends” groups. An advertisement soliciting participants to complete an 10-minute anonymous structured questionnaire was placed on MSM-oriented Tencent QQ “making friends” groups, and potential participants contacted the investigators or the investigators contacted potential participants if they were online. Investigators went into chat rooms and solicited individuals to participate in the survey. Participants were recruited between September 1 and September 30, 2011. The anonymous structured questionnaire completed online was a screening tool that asked for information on age, education level, employment status (full-time or part-time), and self-identified sexual orientation (homosexual, bisexual, heterosexual, or uncertain). If a potential participant wanted to take part in this survey, he was selected to participate in the one-on-one interviews (The participants interviewed on-line).

The protocol was approved by the Ethics Review Committees of the Huzhou Center for Disease Control and Prevention, and all patients provided verbal informed consent to participate in the study. One-on-one interviews on the computer were conducted by three well-trained peer interviewers in September 2011. This cohort was divided into the following two groups: consistent condom users (n = 68) and inconsistent condom users (n = 342). Consistent condom users are MSM who reported using a condom every time with both stable and casual partners, while inconsistent condom users did not. An incentive of RMB 20 Yuan (approximately 3.2 US$) loaded onto a rechargeable card was offered to respondents who completed the interview and provided their phone number. The response rate was approximately 61%, and this figure was defined as the number of completed questionnaires divided by the number of eligible respondents invited to participate in the study.

### Measurements

Information was collected on respondents’ age, education level, employment status (full-time or part-time), and self-identified sexual orientation (homosexual, bisexual, heterosexual, or uncertain). Each respondent was asked whether he had been tested for HIV antibody or received any HIV-related prevention services such as publicity material, condoms and advice in the last 12 months. Eight HIV-related issues were included to assess comprehensive knowledge of HIV among the respondents. The issues included whether an HIV-infected person could look healthy, whether HIV could be transmitted via mosquito bites, whether HIV could be transmitted via sharing a meal with an HIV patient, whether HIV could be transmitted via blood transfusion or using blood products, whether HIV could be transmitted via sharing a needle with an HIV patient, whether HIV could be transmitted to a unborn child via delivery, whether HIV could be transmitted when correctly using a condom every time they have sex, and whether HIV transmission could be reduced by having sex with a steady sexual partner. An individual who responded correctly to more than 5 questions was considered to have comprehensive HIV knowledge.

### Statistical analysis

Bivariate logistic regression analyses were performed to compare the differences between consistent condom users and inconsistent condom users. Variables with significant bivariate between-group differences were used as candidate variables in a stepwise multivariate logistic regression model. The independent variables are listed in Tables 1 and 2, and a summary multivariate model was fitted to identify variables that were independently associated with consistent condom use. All statistical analyses were performed using SPSS for Windows 17.0, and a *p* value < 0.05 was considered to be statistically significant.

**Table 1:** Consistent condom use by socio-demographic characteristics of the respondents

**Variables**	**All (*n*** **= 410)**	**Consistent condom use**		**Univariate *OR *** **( *95% CI *)**
		**Yes (*n*** **= 68)**	**No (*n*** **= 342)**	
	***n *** **( *Col% *)**	***n *** **( *Row% *)**	***n *** **( *Row% *)**	***(Group I vs. Group V)***
Socio-demographics				
**Age groups**				
15-25	227 (55.4)	35 (15.4)	192 (84.6)	1.00
≥26	183 (44.6)	33 (18.0)	150 (82.0)	1.21 (0.72-2.03)
**Education level**				
≤ High school	147 (35.9)	16 (10.9)	131 (89.1)	1.00
≥College/university	263 (64.1)	52 (19.8)	211 (80.2)	2.02 (1.11-3.68)^*^
**Employment status**				
Employed full-time	316 (77.1)	50 (15.8)	266 (84.2)	1.00
Not employed full-time	94 (22.9)	18 (19.1)	76 (80.9)	1.26 (0.69-2.29)
**Marriage**				
Unmarried or divorced	305 (74.4)	53 (17.4)	252 (82.6)	1.00
Married or cohabitating	105 (25.6)	15 (14.3)	90 (85.7)	0.79 (0.43-1.48)
**Had sex with steady male partners**				
No	275 (67.1)	48 (17.5)	227 (82.5)	1.00
Yes	135 (32.9)	20 (14.8)	115 (85.2)	0.82 (0.47-1.45)
**Self-identified as exclusively homosexual**				
Yes	163 (39.8)	29 (17.8)	134 (82.2)	1.00
No (bisexual/not certain)	247 (60.2)	39 (15.8)	208 (84.2)	0.87 (0.51-1.47)
HIV-related prevention services (last 12 months)				
**Tested for HIV**				
No	364 (88.8)	57 (15.7)	307 (84.3)	1.00
Yes	46 (11.2)	11 (23.9)	35 (76.1)	1.69 (0.81-3.53)
**Received other HIV prevention services**				
No	221 (53.9)	31 (14.0)	190 (86.0)	1.00
Yes	189 (46.1)	37 (19.6)	152 (80.4)	1.49 (0.89-2.52)
**Comprehensive knowledge of HIV**				
No	135 (32.9)	9 (6.7)	126 (93.3)	1.00
Yes	275 (67.1)	59 (21.5)	216 (78.5)	3.82 (1.83-7.98)^**^
**Perceived condom efficacy for HIV prevention**				
Low/unknown	59 (14.4)	13 (22.0)	46 (78.0)	1.00
High	351 (85.6)	55 (15.7)	296 (84.3)	0.66 (0.33-1.30)
**Fear of getting HIV via MSM sex behaviors**				
Yes	262 (63.9)	46 (17.6)	216 (82.4)	1.00
A little/no	148 (36.1)	22 (14.9)	126 (85.1)	0.82 (0.47-1.43)

^*^p < 0.05; ^**^p < 0.01.

**Table 2:** Consistent condom use by HIV-related risk behaviors and self-reported STD in the last 6 months

**Behaviors**	**All(*n*** **= 410)**	**Consistent condom use**		**Univariate *OR*** **( *95% CI *)**
		**Yes (*n*** **= 68)**	**No (*n*** **= 342)**	
	***n *** **( *Col% *)**	***n *** **( *Row% *)**	***n *** **( *Row% *)**	**(Group I vs. Group V)**
Number & type of MSM partner				
**Number of male sexual partners in the last 6 months ( *n *)**				
**≤2**	380 (92.7)	58 (15.3)	322 (84.7)	1.00
**>2**	30 (7.3)	10 (33.3)	20 (66.7)	2.78 (1.24-6.23)^*^
**Had sex with male sex workers**				
No	384 (93.7)	51 (13.3)	333 (86.7)	1.00
Yes	26 (6.3)	17 (65.4)	9 (34.6)	12.33 (5.22-29.15)^**^
**Substance use**				
**Drank alcohol before sex**				
Yes	111 (27.1)	9 (8.1)	102 (91.9)	1.00
No	299 (72.9)	59 (19.7)	240 (80.3)	2.79 (1.33~5.83)^**^
**Self-reported contraction of STD**				
No	316 (77.1)	59 (18.7)	257 (81.3)	1.00
Yes	94 (22.9)	9 (9.6)	85 (90.4)	0.46 (0.22~0.97)^*^
**Sought male sexual partners via the internet**				
No	209 (51.0)	28 (13.4)	181 (86.6)	1.00
Yes	201 (49.0)	40 (20.0)	161 (80.0)	1.61 (0.95~2.72)

^*^p < 0.05; ^**^p < 0.01.

## Results

### Background characteristics

Results are summarized in Table 1. Among the respondents, (mean ± standard deviation) of age were 25.5 ± 4.5 years, with the minimal and maximal age of participants was 15 and 47, respectively. In terms of education, 64.1% of had completed college/university or had more education, and the others had not completed college/university. Among those with a college/university education or greater, 19.8% were consistent condom users, while 10.9% of respondents with less than a college/university education were consistent condom users, and the chi-square analysis showed that there was a significant difference (*OR* = 2.02, 95% *CI* = 1.11-3.68, *P* < 0.05) between these two groups.

21.5% of respondents in “had a comprehensive knowledge of HIV” group used condoms consistently, and 6.7% of participants in the comparable group used condoms consistently. The chi-square analysis indicated that there were significant difference (*OR* = 3.82, 95% *CI* = 1.83-7.98, *P* < 0.01) between two groups. Most (85.6%) respondents believed that condoms were highly effective for HIV prevention. Over one-third (36.1%) of respondents had a little or no fear of contracting HIV via MSM sexual behaviors, while the remainder were fearful of contracting HIV via these behaviors.

### Risk behaviors and self-reported STD in the last 6 months

Table 2 shows that 30 participants had more than two male sexual partners, Among these respondents, 33.3% were consistent condom users, the chi-square analysis showed significant difference (*OR* = 2.78, 95% *CI* = 1.24-6.23, *P* < 0.05) compared with the corresponding group. Few respondents (26) reported having sex with male sex workers, 65.4% of these participants reported being consistent condom users, which showed a significant difference (*OR* = 12.33, 95% *CI* = 5.22-29.15, *P* < 0.01) in the chi-square analysis compared with the corresponding group.

Approximately three-quarters of respondents (299) reported that they hadn’t drunk alcohol before sex, 19.7% of these respondents were consistent condom users and a significant difference (*OR* = 2.79, 95% *CI* = 1.33-5.83, *P* < 0.01) were observed in the chi-square analysis. Additionally, 94 respondents reported that they had contracted an STD, 9.6% of these participants were consistent condom users, and the chi-square analysis showed that there was a significant difference (*OR* = 0.46, 95% *CI* = 0.22-0.97, *P* < 0.05) compared with the corresponding group. Nearly half respondents (201) had sought male sexual partners via the internet, and 20.0% of these respondents were consistent condom users, which showed a significant difference (*OR* = 1.61, 95% *CI* = 0.95-2.72, *P* > 0.05) in the chi-square analysis compared with respondents who had not sought partners via the internet.

### A multivariate summary model with respondents

We bring the variables which *P* < 0.1 of the Chi-square analysis (Tables 1 and 2) into the multivariate logistic regression model. The results showed that the consistent condom use rate was higher among the respondents who had a comprehensive HIV knowledge than who did not have it. (*OR* = 4.08, *95% CI*: 1.85-8.99, *P* < 0.01). In addition, those who had sex with male sex workers or those who had not drunk alcohol before sex were more likely to be consistent condom users (*OR* = 15.30, *95% CI*: 5.89-39.75, *P* < 0.01; and *OR* = 3.10, *95% CI*: 1.38-6.95, *P* < 0.01, respectively).

## Discussion

The places of MSM meet up include public toilets, bathrooms, bars and so on. and the way of MSM mostly meet up in China were “for-one-night” (just named as 419) through the Internet such as QQ group or from friend’s introduction. Respondents who were self-reported MSM and chat with other MSM or seek sexual partners of MSM by the Internet would be characterized in comparison to other MSM (venue-based sampling), such as these people may sociable, have an agile mind, and would like to try new things. Our study supports that online behavioral research among MSM is feasible and productive for gathering relevant data [3,17-21]. Our data suggest that among the MSM population, those with a comprehensive knowledge of HIV were more likely to be consistent condom users than those without a comprehensive knowledge, that those who had sex with male sex workers were more likely to be consistent condom users than those who did not, and that those who had not consumed alcohol before sexual contact were more likely to be consistent condom users than those who had consumed alcohol before sex.

Consistent with the results of previous studies [22,23], our research implies that those with comprehensive knowledge of HIV were more likely to use condoms consistently compared with those who did not demonstrate a comprehensive knowledge of HIV. Increasing comprehensive knowledge of HIV along with intensifying efforts to improve safe sexual practices among MSM should be components of MSM-based prevention programs. Prevention strategies that address social acceptability of condoms and social skills related to condom negotiation among MSM are also needed [23].

The findings of this study also show that MSM who make commercial sex visits may use condoms consistently. This may provide an entry point for HIV prevention intervention with men who patronize male sex workers, and such efforts should tap into existing dynamics of social interaction to promote pro-condom norms [24]. Future HIV/AIDS prevention interventions that address consistent condom use among male sex workers should promote educational efforts that focus on awareness of the enduring negative health consequences of acquiring HIV/AIDS, and these interventions should cultivate positive attitudes toward the efficacy of condom use by using creative social marketing strategies [15].

This study attempted to distinguish the socio-cultural profiles of MSM who were consistent condom users with both stable and casual partners and those who were not consistent users. Compared to their counterparts, consistent condom users were less likely to drink alcohol before sex. As we know, alcohol impairs judgment and may be causally implicated in sexual risk taking [25]. Heavy drinking is associated with risky sexual behaviors [26]. Our data suggest that alcohol consumption does decrease consistent condom use, and although the data indicate an association, they do not reveal causation. Improving knowledge of HIV and other STD transmission among sexual partners and challenging expectations of alcohol as a sexual disinhibitor could help decrease the spread of HIV and other STDs [27].

The results of this study indicate that there were no significant differences in education level or the rates of receiving other HIV prevention services, such as receiving publicity material, condoms or advisory services. However, HIV prevention services that aim to reduce the risk of acquiring and transmitting infections among MSM and link infected MSM to treatment must be expanded to reduce HIV infections that are primarily spread through UAI [28]. Sex education programs are effective at increasing HIV/AIDS knowledge and condom use [29]. Future research on the causal mechanisms underlying the association between education and HIV/AIDS prevention should focus on how more advanced schooling enhances the cognitive skills needed for health reasoning [30].

Some limitations of this study should be discussed. Firstly, because of the response rate was low, the representativeness of the study is limited. As many previously published studies of MSM had used similar recruitment methods [16,31], the convenience sampling used here was an improper means of random sampling, which may limit our ability to generalize our findings to other MSM populations [32]. Secondly, accurate alcohol measurement is notoriously difficult. Respondents may reply to different answers, based on a perceived bias in the points of “Drank alcohol before sex”. In addition, although it is frequently used in most sexual behavior studies [3,16,33,34], self-reported data may be subject to potential reporting biases including recall bias social desirability bias. In our studies, respondents were assured of strict anonymity and privacy of the interviews to reduce reporting biases. Finally, biological markers such as HIV testing were not collected in this study, so we know little about the HIV status of these individuals. HIV-positive individuals may have higher levels of risky behavior compared to their HIV-negative counterparts. Further research is needed to prove this hypothesis. Estimates of high-risk sexual behavior based on Internet convenience samples are also likely to overestimate the levels of risky sexual behaviors in the wider MSM population [35].

## Conclusions

The findings of this study have some implications for the HIV-related risky behaviors of MSM who use condoms consistently with both stable and casual partners. The groups of consistent condom users in this study had a comprehensive knowledge of HIV, were more likely to have ever had sex with male sex workers and were more likely to not consume alcohol before sex. Increasing comprehensive knowledge of HIV among sexually active MSM should contribute to HIV prevention. Increasing correct and consistent condom use among sexually active adolescents remains a critical public health goal [36], and MSM may serve as the main population to achieve this goal through sexuality education and the encouragement of consistent condom use. More public health education should focus on the MSM population via the Internet.

## Abbreviations

HIV: Human immunodeficiency virus; MSM: Men who have sex with men; UAI: Unprotected anal intercourse; STD: Sexually transmitted diseases.

## Competing interests

The authors declare that they have no competing interests.

## Authors’ contributions

JM, HJ and DZ participated in the design of the study and data collection. YZ performed the statistical analysis. YZ and JM conceived of the study and participated in its design and coordination and helped to draft the manuscript. All authors read and approved the final manuscript.

## Pre-publication history

The pre-publication history for this paper can be accessed here:

http://www.biomedcentral.com/1471-2458/13/1101/prepub
